# Supplementary material for: COZOID: contact zone identifier for visual analysis of protein-protein interactions
Source: BMC Bioinformatics. 2018 Apr 6;19:125. doi: 10.1186/s12859-018-2113-6 (PMC5889581; doi:10.1186/s12859-018-2113-6)
Supplement: Supplementary file 2 — Software build. Executable binary file of the software tool. (ZIP 118,784 kb) [file 12859_2018_2113_MOESM2_ESM.zip › cozoid/java/docs/beansbinding-1.2.1-doc.zip!/org/jdesktop/el/MethodInfo.html]

MethodInfo (Beans Binding)


---


|  |  |  |
| --- | --- | --- |
| **Overview** \| **Package** \| **Class** \| **Use** \| **Tree** \| **Deprecated** \| **Index** \| **Help** | |  |
| **PREV CLASS**   **NEXT CLASS** | **FRAMES**    **NO FRAMES**     **All Classes** |  |
| SUMMARY: NESTED \| FIELD \| CONSTR \| METHOD | DETAIL: FIELD \| CONSTR \| METHOD |  |


---


## org.jdesktop.el Class MethodInfo

```
java.lang.Object
  org.jdesktop.el.MethodInfo
```

---

``` public class MethodInfo extends java.lang.Object ```

Holds information about a method that a `MethodExpression`
evaluated to.

**Since:**
:   JSP 2.1

---

**Constructor Summary**

`MethodInfo(java.lang.String name,
java.lang.Class<?> returnType,
java.lang.Class<?>[] paramTypes)`
  
          Creates a new instance of `MethodInfo` with the given
information.


| **Method Summary** | |
| --- | --- |
| `java.lang.String` | `getName()`             Returns the name of the method |
| `java.lang.Class<?>[]` | `getParamTypes()`             Returns the parameter types of the method |
| `java.lang.Class<?>` | `getReturnType()`             Returns the return type of the method |

**Methods inherited from class java.lang.Object**

`clone, equals, finalize, getClass, hashCode, notify, notifyAll, toString, wait, wait, wait`

**Constructor Detail**

### MethodInfo

```
public MethodInfo(java.lang.String name,
                  java.lang.Class<?> returnType,
                  java.lang.Class<?>[] paramTypes)
```

:   Creates a new instance of `MethodInfo` with the given
    information.

    **Parameters:**: `name` - The name of the method: `returnType` - The return type of the method: `paramTypes` - The types of each of the method's parameters


**Method Detail**

### getName

```
public java.lang.String getName()
```

:   Returns the name of the method

    :   **Returns:**: the name of the method

---


### getReturnType

```
public java.lang.Class<?> getReturnType()
```

:   Returns the return type of the method

    :   **Returns:**: the return type of the method

---


### getParamTypes

```
public java.lang.Class<?>[] getParamTypes()
```

:   Returns the parameter types of the method

    :   **Returns:**: the parameter types of the method


---


|  |  |  |
| --- | --- | --- |
| **Overview** \| **Package** \| **Class** \| **Use** \| **Tree** \| **Deprecated** \| **Index** \| **Help** | |  |
| **PREV CLASS**   **NEXT CLASS** | **FRAMES**    **NO FRAMES**     **All Classes** |  |
| SUMMARY: NESTED \| FIELD \| CONSTR \| METHOD | DETAIL: FIELD \| CONSTR \| METHOD |  |


---
